# Patient reported outcomes can improve performance status assessment: a pilot study

**DOI:** 10.1186/s41687-019-0136-z

**Published:** 2019-07-16

**Authors:** Joan E. Broderick, Marcella May, Joseph E. Schwartz, Ming Li, Aaron Mejia, Luciano Nocera, Anand Kolatkar, Naoto T. Ueno, Sriram Yennu, Jerry S. H. Lee, Sean E. Hanlon, Frankie A. Cozzens Philips, Cyrus Shahabi, Peter Kuhn, Jorge Nieva

**Affiliations:** 1 Dornsife Center for Self-Report Science, University of Southern California, 635 Downey Way, Los Angeles, CA 90089-3332 USA; 2 Department of Psychiatry, Stony Brook University, Stony Brook, USA; 3 Norris Cancer Center, University of Southern California, Los Angeles, USA; 4 Department of Computer Sciences, University of Southern California, Los Angeles, USA; 5 Department of Biological Sciences, University of Southern California, Los Angeles, USA; 6 Department of Breast Medical Oncology, The University of Texas MD Anderson Cancer Center, Houston, USA; 7 Morgan Welch Inflammatory Breast Cancer Research Program and Clinic, The University of Texas MD Anderson Cancer Center, Houston, USA; 8 Department of Palliative Care Medicine, The University of Texas MD Anderson Cancer Center, Houston, USA; 9 Center for Strategic Scientific Initiatives, National Cancer Institute, Bethesda, USA

**Keywords:** Performance status, Actigraphy, Patient reported outcomes, Body weight, Oncology

## Abstract

**Background:**

Patient performance status is routinely used in oncology to estimate physical functioning, an important factor in clinical treatment decisions and eligibility for clinical trials. However, validity and reliability data for ratings of performance status have not been optimal. This study recruited oncology patients who were about to begin emetogenic palliative or adjuvant chemotherapy for treatment of solid tumors. We employed actigraphy as the gold standard for physical activity level. Correspondences between actigraphy and oncologists’ and patients’ ratings of performance status were examined and compared with the correspondences of actigraphy and several patient reported outcomes (PROs). The study was designed to determine feasibility of the measurement approaches and if PROs can improve the accuracy of assessment of performance status.

**Methods:**

Oncologists and patients made performance status ratings at visit 1. Patients wore an actigraph and entered weekly PROs on a smartphone app. Data for days 1–14 after visit 1 were analyzed. Chart reviews were conducted to tabulate all unexpected medical events across days 1–150.

**Results:**

Neither oncologist nor patient ratings of performance status predicted steps/hour (actigraphy). The PROMIS® Physical Function PRO (average of Days 1, 7, 14) was associated with steps/hour at high (for men) and moderate (for women) levels; the PROMIS® Fatigue PRO predicted steps for men, but not for women. Unexpected medical events occurred in 57% of patients. Only body weight in female patients predicted events; oncologist and patient performance status ratings, steps/hour, and other PROs did not.

**Conclusions:**

PROMIS® Physical Function and Fatigue PROs show good correspondence with steps/hour making them easy, useful tools for oncologists to improve their assessment of performance status, especially for male patients. Female patients had lower levels of steps/hour than males and lower correlations among the predictors, suggesting the need for further work to improve performance status assessment in women. Assessment of pre-morbid sedentary behavior alongside current Physical Functioning and Fatigue PROs may allow for a more valid determination of disease-related activity level and performance status.

## Introduction

When treating cancer patients, oncologists routinely utilize performance status (PS) scales to measure an individual’s ability to engage in physical activities and care for self. PS is designed to fulfill crucial roles in cancer treatment, serving as an indicator of prognosis and one determinant of patient eligibility for clinical trials [2]. The two most commonly used scales are the Karnofsky Performance Status (KPS) scale [13] and the Eastern Cooperative Oncology Group (ECOG) scale [18, 33] (ordinal ratings of 0 = healthy, 1, 2, 3, 4, 5 = deceased) derived from the KPS. Both were introduced in the post-World War II years in efforts to standardize data collection in clinical oncology trials. Over 60 years later, the observed psychometric characteristics of the scales suggest the need for an updated approach. Numerous studies evaluating the scales have found them to be limited in terms of both reliability and validity.

Interrater reliability indicates the degree to which ratings from two or more raters are in agreement with one another. In oncology, low interrater reliability of PS ratings is common. Ando examined interrater reliability for ECOG ratings in 206 patients with advanced non-small cell lung cancer [1]. Oncologists, patients, and nurses provided ratings, and there were significant differences among the three groups’ assessments. Agreement among the groups was typically moderate (weighted *k* = 0.53, 0.63, and 0.56, respectively), with oncologists tending to give the healthiest ratings and patients the poorest.

Sørensen conducted similar research comparing physician-physician (*n* = 3) ECOG interrater reliability in a group of 100 patients with heterogeneous tumor sites [28]. Half of the patients were receiving chemotherapy or radiation therapy, and 40% had ECOG scores of 2 or greater. What is generally interpreted as low-moderate agreement was observed among the three raters (*k* = 0.44); < 0.40 is considered poor [9]. The two healthiest performance status categories (0–1) yielded somewhat better agreement (*k* = 0.55 and 0.48, respectively) compared with categories 2–4 (*k* = 0.43 to 0.31). These findings of low-moderate reliability have been observed consistently across many other studies, e.g., Blagden [2] and Taylor [29]. Furthermore, poor reliability of PS scores has been associated with poor clinical outcomes. In a study of 1636 patients with advanced lung or colorectal cancer, patients and physicians disagreed on PS scores more than half of the time (weighted *k* = 0.30 and 0.35, respectively), and disagreement between their KPS ratings was associated with significantly shorter duration of survival (average 9.4 vs. 11.8 months) [26].

Similarly, research examining the validity of PS scores has yielded modest results. Of concern, most relevant studies have examined validity in groupings of ECOG scores (e.g., 0–2 v. 3–4) that do not allow for tests of validity across each PS level. For example, Quinten reported a meta-analysis of 30 randomized controlled trials from the European Organization for Research and Treatment of Cancer [21]. Data covered 1986–2004, and trials included 10,108 patients with 11 different cancer sites. Poor ECOG was found to significantly predict survival (*HR* = 3.09, *p* < .0001). However, the dichotomization of ECOG that was employed into good (0–1) and poor (2–3) PS constitutes a loss of detailed information for each level; and, due to clinical trial eligibility criteria, 88% of the sample fell into the former group. The limited attention paid to variability within the upper and lower levels of PS is problematic, as clinical decisions are often made at the more granular level. Finally, emerging evidence suggests that more objective measures of performance status can improve validity over subjective measures. In pilot work, Pirl and colleagues found that although both ECOG and actigraphy were predictive of survival in stage IV non-small cell lung carcinoma patients, only actigraphy discriminated differences in survival in patients with good PS (ECOG 0–1) [19].

Given the utilization of PS scores in cancer treatment, inaccuracies can have negative consequences. For example, patients can be precluded from potentially helpful treatment or, conversely, subject patients to treatments that are too aggressive, given their physical condition. Additionally, unreliable ratings can result in patients with truly poor PS entering clinical trials, thereby increasing the number of trial “failures” and limiting comparability and generalizability across studies.^6^ Determining an ECOG rating is generally done by observing a patient’s ambulation in clinic, review of medical history, and queries during consultation. While reasonable, it is constrained by contextual factors that may not generalize to a patient’s activity outside of clinic due to the lack of systematic assessment in the patient’s natural environment. These problems underlie the need for an improved assessment approach to characterize PS.

Recent developments suggest that new technologies have the potential to provide standardized and objective assessments of patients’ PS that could increase the precision of clinical decision-making and clinical trial enrollment eligibility. Among these are objective activity measures, such as actigraphy, and self-reported, brief, validated patient reported outcomes (PROs) that are much easier to collect than actigraphy data. This pilot-feasibility study examined the correspondence of PROs with actigraphy to determine the criterion validity of PROs to reflect activity level to assist in PS determination.

## Methods

### Recruitment and participants

Patients (*N* = 65) were recruited at four clinical sites: University of Southern California Norris Cancer Hospital, USC-Los Angeles County Hospital, Hoag Hospital Newport Beach, and The University of Texas MD Anderson Cancer Center. Inclusion criteria were age ≥ 18, plans for at least 2 planned cycles of highly emetogenic palliative or adjuvant chemotherapy for treatment of solid tumor, and the ability to ambulate without assistive devices. Exclusion criteria included missing limbs, symptomatic brain metastasis, or known movement disorders including essential tremor requiring drug therapy. Tumor sites of recruited patients included head and neck, breast, bladder, thymoma, testicular, lung, gallbladder, and adrenal cancers. All recruited patients signed written consent to engage in the protocol requiring recording of symptoms on a phone app and wearing an activity tracker.

### Procedures

This was a single-arm, observational study conducted between May 2016 – May 2017. Potentially eligible patients were presented with the opportunity to participate in the study at their oncology consultation by clinical research coordinators. Interested patients reviewed the study consent form that was approved by the Institutional Review Boards of each participating hospital. Patients were enrolled in the study either at the consultation visit or the following clinic visit and were provided with a Microsoft Band 2 (actigraphy), a Microsoft Lumia 550 Windows smartphone with study app installed, and a Utopia digital scale to measure body weight. Patients were gifted these devices at the end of the study.

Patients were asked to wear the Microsoft Band during the day and to open the study app on the study phone each evening to record symptoms over the 60 days of the protocol. PROs were available between 6 pm and 12 am, and patients received a reminder to complete the PROs via text message sent to the study phone between 7 and 8 pm. PROs remained available throughout the 6-h period, and patients were able to exit out of the app and continue where they left off upon re-entering the app. Patients were asked to sync the Microsoft Band with the study phone by opening the Microsoft Health app and to charge the Microsoft Band and study phone each evening.

During the 60-day protocol, patients received at least two highly emetogenic chemotherapy cycles as prescribed individually by their oncologist. The type and schedule of administration was not determined by the study protocol, thus reflecting the idiosyncratic treatment decisions of patient and oncologist and increasing generalizability of findings.

### Materials

#### Activity tracker

In oncology, performance status (PS) is designed to reflect the physical capacity and functioning of a patient. For example, a score of 2 on the ECOG is defined as “Ambulatory and capable of all selfcare but unable to carry out any work activities; up and about more than 50% of waking hours.” [18]. In this study, actigraphy is considered the objective “gold standard” for assessing patients’ physical activity [30], and use of step count has been recommended as an improved performance status measure in cancer care [20]. The Microsoft Band, a wrist-worn fitness tracker similar to a FitBit, was used in this study to measure steps. These types of trackers have demonstrated moderate to strong validity in relation to research grade accelerometers for step counts [7, 17, 31], and the Band specifically performs comparably to the trackers examined in validity trials [27]. Patients were asked to wear the Microsoft Band each day during their waking hours throughout the 60-day protocol. The Microsoft Band generated hourly data on heart rate, calories expended, and step counts; these data were automatically delivered to the study server. No data were retained on the Band.

Although daily data was collected across 60 days of the protocol, this report summarizes daily step counts for Days 1–14 of the protocol as this time frame would be most relevant for the oncologist’s initial assessment of PS. Many patients received a chemotherapy infusion in Days 1–7; in most cases there was no infusion in Days 8–14. Thus, the average step count for each patient captured activity during infusion and non-infusion periods to best characterize their activity levels. In order to maximize reliability of daily step counts, days with less than 6 h of Band wear were excluded. Only full hours of wear were included in the analyses. Total steps/day during complete wear hours were summed across the 14 days and divided by the total number of full-wear hours. Across patients, over 86% of days yielded the required ≥6 full hours of wear time.

#### Daily and weekly patient reported outcomes (PROs) and body weight

The app installed on the Microsoft phones was created and tested by engineers on the study team. The app collected patient responses to standardized PROMIS® PRO items measuring physical function, fatigue, sleep disturbance, social isolation, and a single item measure of appetite (see: *http://www.healthmeasures.net/index.php?option=com_content&view=category&layout=blog&id=147&Itemid=806**).* Body weight from scale measurement was also recorded.

Three language versions were available to match the needs of the recruited sample: English, Spanish, and Traditional Mandarin. Official PROMIS® translations for Spanish were used for Fatigue, Physical Function, Sleep Disturbance and Social Isolation scales as well as PROMIS® translations for Traditional Mandarin for Fatigue, Physical Functioning and Sleep Disturbance scales. Translation of the Social Isolation scale for Mandarin and the appetite item (Spanish and Mandarin) were conducted using forward and back-translation methods by native speakers as recommended by PROMIS® methods (see http://www.healthmeasures.net/explore-measurement-systems/promis/intro-to-promis/available-translations).

PROMIS® short forms measuring physical function (v1.0, 10 items) [23], fatigue (v1.0, 7 items) [5], and sleep disturbance (v1.0, 8 items) [32] were administered on Days 1, 7, and 14 using a 7-day recall period. Social isolation (v2.0 6 items) [10] was measured only on Day 1. These scales use 5-point Likert response options of frequency (“never” to “always”) or intensity (“not at all” to “very much”) as appropriate for the construct. PROMIS® scales are scored in the direction of more of the construct, i.e., a higher fatigue score indicates more fatigue. They also are constructed using a *T*-score metric where 50 is the mean of a relevant reference population, and 10 is the standard deviation (*SD*) of that population. In most cases the reference group for *t*-scores is the general U.S. adult population, as was the case for the scales used in this study. These scales have demonstrated good validity and reliability in a variety of clinical groups including cancer [4, 5, 25].

Patients were additionally asked each day to respond to a single item of appetite modified for daily use from the Symptom Distress Scale [15] (“Consider how your appetite was today, and select the item that best describes it”: 1- “My appetite was normal and I could enjoy good food”, 2- “My appetite was somewhat less than normal”, 3- “I didn’t really enjoy my food”, 4- “I had to force myself to eat my food” "5- “I could not stand the thought of food”). This item has demonstrated good validity using Item Response Theory analysis [4]. Patients also entered daily their body weight in pounds as measured by the study scale, an average was computed of these weights across days 1–14. *T*-scores were used for the PROMIS® scales while raw scores were used for the appetite item, and body weight. An average scaled score for each PRO for Days 1, 7, and 14 was computed to directly map onto the reporting period of the activity tracker.

#### Performance status ratings

An ECOG score was recorded in the medical chart by patients’ treating oncologists at the patient’s consultation. At this same first visit, patients were asked to independently make an ECOG rating. This single item provides six response options from 0 = healthy to 5 = deceased.

#### Chart review of unexpected medical events

At the conclusion of the study, patient charts were reviewed by the study oncologist (JN) for unplanned healthcare encounters across the 60-day protocol and subsequent 90 days of follow-up (total 150 days). Unexpected medical events were clinic, urgent care, day hospital, and hospital admissions related to the cancer diagnosis that were outside of scheduled clinic and infusion visits. Routine office visits, planned chemotherapy hospitalizations, and planned surgery hospitalizations were not included.

### Analysis

Data were analyzed with SAS and STATA. Pearson and Spearman correlations were conducted to evaluate the magnitude of the associations of predictors with outcomes. Given the relatively small sample size for this pilot study, instead of traditional significance testing that reports probability levels, we present statistics with confidence intervals so the reader may judge the precision of the estimates. To help with interpretation, confidence intervals that do not include zero would be considered significant.

## Results

A total of 65 patients were consented for the study to initiate the screening process. Of these, 16 did not meet eligibility criteria and an additional 7 patients were withdrawn from the study due to geographic relocation, non-compliance with daily use of the devices, and visual difficulties that made use of the study app difficult. This yielded data from 42 patients for analysis, though some analyses have smaller N’s due to missing data. Demographic and clinical characteristics of the analyzed patients’ data are shown in Table 1.

**Table 1:** Demographic and clinical descriptors of analyzed sample

Sex	
Males	50%, *N* = 21
Females	50%, *N* = 21
Age (M, *SD,* range*)*	
Males	43.6 (11.6), 24–66
Females	52.8 (11.7), 37–72
Race/Ethnicity	
White	91%, *N* = 38
Black	5%, *N* = 2
Asian	5%, *N* = 2
Hispanic	55%, *N* = 23
Education	
0–8 yrs	12%, *N* = 5
9–11 yrs	12%, *N* = 5
High school grad	21%, *N* = 9
Some college	14%, *N* = 6
College grad	38%, *N* = 16
Marital Status	
Married	55%, *N* = 23
Single	26%, *N* = 11
Divorced	14%, *N* = 6
Widowed	5%, *N* = 2
Employed	
Yes	19%, *N* = 8
No	81%, *N* = 34
Recruitment Site	
LA/USC Medical Center	60%, *N* = 25
USC Norris Cancer Center	19%, *N* = 8
MD Anderson Cancer Center	19%, *N* = 8
Hoag Hospital	2%, *N* = 1
Primary Cancer Site	
Breast	40%, *N* = 17
Testicular	24%, *N* = 10
Head and Neck	17%, *N* = 7
Bladder	5%, *N* = 2
Lung	5%, *N* = 2
Thymoma	5%, *N* = 2
Adrenal	2%, *N* = 1
Gallbladder	2%, *N* = 1
Cancer Stage	
No evidence of disease	7%, *N* = 3
Locally recurrent	33%, *N* = 14
Distant metastasis	50%, *N* = 21
Physician ECOG	
Level 0	54%, *N* = 22
Level 1	44%, *N* = 18
Level 2	2%, *N* = 1
Patient ECOG	
Level 0	34%, *N* = 11
Level 1	50%, *N* = 16
Level 2	13%, *N* = 4
Level 3	3%, *N* = 1
PROs^a^ (M, SD, range)	
Physical Function	43.9 (7.0), 29–62
Fatigue	53.1 (8.1), 34–66
Sleep Disturbance	51.5 (7.6), 30–69
Social Isolation	46.1 (7.1), 34–62

Rounding and missing data account for sums not always equal to 100

^a^General population PROMIS® scale *t*-scores have mean = 50, *SD* = 10; higher scores reflect higher level of construct

Observed oncologist ECOG ratings were evenly divided into levels 0 and 1; only one patient was rated as 2. Patient ECOG ratings ranged from 0 to 3 and were higher (poorer PS) than the oncologist ratings (see Table 1). Mean PROMIS® PRO scores for the sample indicate that compared to the general adult population patients reported less physical function, (− 0.6 *SD*), more fatigue (+ 0.3 *SD*), more sleep disturbance (+ 0.2 *SD*), and less social isolation (− 0.4 *SD*). Knowing that PROMIS scores have a standard deviation of 10, a − 0.6 *SD* difference for physical function can be interpreted as a percentile rank of about 30 relative to 50 for the population mean (*SD = 10)*, meaning that 70% of the general population would have greater physical function than our patients.

As would be expected, men and women subgroups had different mean aggregated body weight measured across Days 1–14. Women, who on average were 10 years older than the men, had a distribution that included more who were overweight and obese (*M* = 166.2 lbs., *SD* = 32.5, range = 120–264). The men’s distribution showed fewer overweight and obese patients (*M* = 176.2 lbs., *SD* = 37.1, range = 120–229).

Over the 42 patients and across the 14 days of data considered in analyses, overall compliance with complete nightly submission of PROs was 88% (*SD* = 17%; median: 93%, range: 7%–100%). One of the participants refused to enter her weight, and technological difficulties accounted for 5 other instances of missing data. Discounting all instances caused by technological difficulties as well as those instances in which only weight data were missing, the compliance rate was 90% (*SD* = 13%, median: 93%, range: 33% - 100%).

### Predicting activity tracker average steps/hour

Age predicted average steps/hour (*r* = −.43, *N* = 35, CI: −.67 to −.11) with greater activity recorded in younger patients. Visual inspection of the steps data revealed one male patient with a very high number of steps/hour (1230, *Z* = 3.70), and we consider this individual an outlier. The association between age and steps with the outlier removed is considerably reduced, −.32 (*N* = 34; CI: −.60 to .02). This outlier patient was removed from subsequent analyses. Steps (outlier removed) also varied by sex (*t* = − 2.07, *N* = 34, *p* = .047): females had an average of 238 steps/hr. (*N* = 19, SD: 152.7, CI: 164 to 311), whereas males had 369 (*N* = 15, SD: 214.0, CI: 250 to 487). Based on this sex difference in steps, we present predictor relationships for the whole sample and by sex.

#### Predicting steps: ECOG

Neither oncologist nor patient ECOG ratings corresponded well to steps/hr. looking at the whole sample and for each sex (see Table 2). Correlations ranged only from −.08 to −.38 with all confidence intervals including zero.

**Table 2:** ECOG predicting steps/hour

ECOG Rater	Overall	Males	Females
Oncologist	*r*_*s*_ *= −.28* *N* = 33, CI: −.57 to .07	*r*_*s*_ = −.38 *N* = 15, CI: −.75 to .17	*r*_*s*_ = −.29 *N* = 18, CI: −.67 to .20
Patient	*r*_*s*_ *= −.08* *N* = 26, CI: −.46 to .32	*r*_*s*_ = −.09 *N* = 9, CI: −.71 to .61	*r*_*s*_ = −.13 *N* = 17. CI: −.58 to .37

These are Spearman correlations

#### Predicting steps: PROs

The Physical Function PRO demonstrated a moderate association (*r* = .57) with steps/hr. for the full sample (see Table 3). This was also observed within both male (*r* = .75) and female (*r* = .57) patients, where a worse Physical Function score corresponded to fewer steps/hr. For male patients only, the Fatigue PRO also showed high correspondence with steps/hr. (*r* = −.79) with greater Fatigue associated with fewer steps.

**Table 3:** PROs predicting average steps/hour

PRO	Overall	Males	Females
Physical Function	***r =*** **.57** ***N*** **= 34, CI: .29 to .76**	***r*** **= .75** ***N*** **= 15, CI: .38 to .91**	***r*** **= .57** ***N*** **= 19, CI: .16 to .81**
Fatigue	*r =* −.39 N = 34,CI: −.65 to −.07	***r*** **= −.79** ***N*** **= 15, CI: −.93 to − .47**	*r* = −.18 *N* = 19, CI: −.58 to .30
Sleep Disturbance	*r =* −.04 N = 34, CI: −.37 to .31	*r* = −.07 *N* = 15, CI: −.56 to .46	*r* = −.15 *N* = 19, CI: −.57 to .33
Appetite Loss	*r =* −.17 N = 34, CI: −.48 to .18	*r* = −.33 *N* = 15, CI: −.72 to .22	*r* = −.15 *N* = 19, CI: −.57 to .33
Social Isolation	*r =* −.03 N = 34, CI: −.37 to .31	*r* = .04 *N* = 15, CI: −.48 to .54	*r* = −.13 *N* = 19, CI: −.55 to .35

These are Pearson correlations. Bold face identifies correlations whose confidence interval does not include zero

### Predicting unexpected medical events

Unexpected medical events were observed in 57% of the patients. Twelve patients had 1 event and 12 patients had ≥2 events.

#### ECOG

Both physicians’ and patients’ ECOG ratings were not correlated with unexpected medical events (r_s_s < .15).

#### Average steps/hour

Actigraphy (mean steps/hr) did not predict the number of unexpected medical events for the entire sample (*r* = .02, *N* = 33, CI: −.32 to .36) or for male or female subgoups.

#### PROs

Physical Functioning, Fatigue, Sleep Disturbance, and Social Isolation PROs did not predict medical events. For these PROs the correlations were ≤ .11 in the whole sample and in the sex subgroups. The Appetite item also did not predict events where *r* = .23, (CI: − 0.09 to 0.50) for the whole sample, *r* = .01 (CI: − 0.43 to 0.44) for females, and *r* = .34 (CI: − 0.13 to 0.68) for males.

#### Body weight

Body weight was correlated with unexpected medical events (*r* = .36, *N* = 41, CI: .06 to .60). It predicted the number of unexpected medical events for women (*r* = .46, *N* = 21, CI: .03 to .74) where higher body weight (overweight – obesity levels) in women was associated with more events, but less so for men (*r* = .31, *N* = 20; CI: −.16 to .66).

## Discussion

This pilot-feasibility study was designed to evaluate new approaches for assessing patient physical activity to improve or replace PS scores that have demonstrated limited reliability and validity. The study examined patient willingness to wear the Microsoft Band to measure activity on a daily basis and to complete PROs and body weight using the app on the mobile phone given to them. Our primary clinical site was the Los Angeles County Hospital, which primarily serves an ethnically diverse, low-income population facing many life challenges. Nevertheless, compliance with wearing the activity tracker and daily engagement with the PRO app was good. Less than 10% of patients were withdrawn from the study due to difficulties or disinterest in interfacing with the study assessment devices. The patients generated ≥6 h of actigraphy on 86% of the first 14 study days that were used for analysis. Likewise, patients recorded data on the app on 88% of days. These results are very encouraging for implementation of these assessment approaches.

After feasibility, we explored various physician and patient self-report measures for their validity via their associations with physical activity measured with actigraphy, our gold standard. That is, can any of the self-report measures that are comparatively easier to obtain be used in place of actigraphy? Finally, we explored the ability of the self-report measures and actigraphy to predict subsequent unexpected medical events, an outcome important in clinical trials. Given the sample size of this pilot study, we conceptualize these analyses as preliminary and useful for generating hypotheses in future, larger studies.

Originally, PS scales were designed to capture oncologists’ impressions of patient activity level, including overall activity, time in bed, and ability to care for self [13, 33]. In contrast to this subjective assessment, actigraphy can be considered a “gold standard” for objective measurement of patients’ activity in their natural environment. We examined the relationship of oncologists’ and patients’ ECOG ratings and PROs collected during the 14 days following study visit 1 with actigraphy for the same period.

Using steps/hour from actigraphy across Days 1–14 as the gold standard for patient activity level, we examined the criterion validity of oncologists’ and patients’ ECOG scores and aggregated PROs across the same reporting period. Neither oncologists’ nor patients’ ECOG ratings predicted actigraphy with any accuracy. What might explain this? From a measurement perspective, it is not surprising that patient and oncologist ECOG ratings performed poorly. The ECOG measure is a single item with 6 response options, but functionally only 5 options as one is a designation for deceased. The scale is further constrained by little use of the fifth option as it represents patient functioning that is severly impacted and often only observed in patients no longer receiving treatment who are close to death. Thus, the ECOG is an assessment that typicaly only uses 4 response options. In fact, patients were rated by oncologists as having good PS on ECOG (scores 0–1) with only one patient having a physician-scored ECOG of 2 (higher scores indicate worse functioning). This constrained almost all ECOG scores to only two levels. Patient ECOGs had somewhat more variability. As outlined in the introduction, biases in the ECOG assessment exist for both clinician and patient. For example, the increasing necessity that ECOG scores show good PS in order to permit clinical trial participation and to avoid payor and administrator scrutiny over decisions to treat patients (who are more ill) may explain why scores were so good in a group of patients whose actigraphy showed relative inactivity. This may also explain why patient self assessment on the ECOG was generally worse than clinician assessment; they were conveying greater limitations than the oncologists, though still not corresponding well to hourly steps. These scoring constraints and probable biases may have contributed to the lack of association between ECOG scores and actigraphy. Or, the result may indicate that ECOG ratings do not convey patients’ activity level with any accuracy.

Next, we examined the criterion validity of the PROs. We chose PROs that made logical sense as associated with physical activity (Physical Functioning and Fatigue) or cancer disease activity and outcomes (Sleep Disturbance, Social Isolation, Appetite). Results were quite positive with PROMIS® Physical Function and Fatigue scores corresponding at high levels with actigraphy, especially for men. The other PROs did not correspond to actigraphy. These findings may also be driven by measurement issues. We used high quality PROs with multiple items for each relevant construct. The high PRO associations with actigraphy likely benefited from the higher reliability and validity of PROs relative to ECOG. These results suggest that assessment of Performance Status can be improved by augmenting or replacing oncologist ECOG ratings with brief, easily administed PROs of physical functioning and fatigue.

Unexpected medical events are important outcomes, particularly in clinical trials. Performance Status has been used clinically to screen patients for the likelihood of toxicity-related events that result in withdrawing a patient from the trial, though there is scant literature to support this use. Research has shown some predictive ability of ECOG for length of survival [21], but has not examined the predictive validity for toxicity. Nevertheless, since it is used in this way clinically, we examined our predictors for these unexpected medical events. Fifty-seven percent of our patients experienced ≥1 unexpected medical event across the 150 days of the study. Neither oncologist nor patient ECOG ratings predicted the number of events. Likewise, actigraphy across Days 1–14 did not predict events, and none of the PROs assessed at study onset predicted events. Aggregated mean body weight across Days 1–14 did predict the number of events with overweight-obese women experiencing more events than normal weight women. In men, body weight did not predict events. Excess body weight in the general population previously has been noted to be associated with increased risk for hospitalization and other adverse health outcomes [24]. Thus, this finding is not specific to cancer, but nevertheless can be important information for oncologists. The lack of an association with men in our study is likely due to low rates of excess weight in this subgroup.

Thus, none of the measures of physical activity – oncologist and patient ECOGs, PROs, and actigraphy - collected within 2 weeks of study onset predicted unexpected medical events across the subsequent 150 days. This raises the question of whether physical activity at time of consult is actually a useful predictor of events. At the time of initial consultation, treatment planning, and screening for trial eligibility, other predictive factors may turn out to be better predictors of subsequent patient failure in clinical trials than activity level. A composite of parameters including gender, weight and other clinical measures may be required to achieve the better predictions. On the other hand, more complex physical assessments, such as comprehensive geriatric assessments (CGA), have demonstrated the ability to identify patients at risk for increased toxicity (unexpected medical events), and may provide a greater degree of granularity in assessing performance necessary for prediction of toxicity [6].

Because women were less physically active than men on actigraphy, we decided to also look at predictor relationships by sex, particularly in light of the NIH recommendation to do so in biomedical research [16]. Less activity in women was not an anticipated finding as several epidemology studies have not observed significant sex differences in the general population [8, 11, 22]; however, the women in our study were about 10 years older than the men, largely due to the number of enrolled young men with testicular cancer, and activity level does gradually decrease with age. Assessing activity level after the cancer diagnosis conveys current levels, but it does not indicate to what degree the activity level is a function of disease status. Patients could have been more active prior to onset of disease or, conversely, may have been equally sedentary before and after the diagnosis. Assessment of pre-morbid sedentary behavior via a PRO [3] could assist in parsing the patients’ premorbid activity level from the current disease-related levels, thus, improving assessment of disease-related performance status.

This study suggests that even in ethnically diverse, low SES samples, patients’ collection of actigraphy and PRO data is clinically feasible to improve assessment of PS. While actigraphy is the gold standard for assessing physical activity, these data suggest that a highly to moderately accurate assessment of patient activity can be achieved with the 10-item PROMIS® Physical Functioning and 7-item PROMIS® Fatigue PROs. Use of PROs in clinical settings is becoming increasing feasible and common, as they are readily collected at clinic visits on paper or tablets and through patient portals [12, 14]. Given the clinical use of PS for treatment decisions, contemporary approaches to assessment can improve accuracy and increase confidence in clinical decision-making yielding potentially improved outcomes and quality of life. By considering patients’ physical stamina when deciding on the degree of aggressiveness of available treatments, patients can be selected who might tolerate highly aggressive treatment or who might have improved quality of life opting for milder treatment.

Several limitations should be noted. This was a small pilot study for the purpose of examining feasibility and hypothesis generation. Small overall sample size limited power, thus confidence intervals were reported rather than significance testing for the correlations. Results warrant replication in larger samples. Second, PROs were assessed weekly across the protocol, and were averaged to increase reliability across three assessments (Days 1, 7, and 14). In clinic settings, a single assessment at the initial consultation would be most efficient, and subsequent research needs to examine the criterion validity of a single PRO assessment. Third, our use of average step counts per hour may or may not be the most informative metric for characterizing patients’ performance status. For example, actigraphs can also be used to estimate energy expenditure. Various actigraphy metrics should be evaluated empirically to identify the most useful approach in oncology. Fourth, likely due to typical age of onset of some of the sex-specific cancers, on average women were 10 years older than men. Thus, differences in observed outcomes for men and women could be driven by age differences. Fifth, the results from this study with adults may not generalize to pediatric populations. PROMIS® PROs are also available for children and this could be explored. Finally, this study did not include a measure of premorbid sedentary behavior, thus it was not possible to specificially isolate the impact of the disease on physical activity distinct from premorbid activity levels.

## Conclusions

Current methods of clinical assessment of cancer patients’ performace status relies upon limited information gathered during consultation with the oncologist. Numerous studies docment the inadequate reliability and validity of this approach. This study observed high levels of correspondence between actigraph-generated steps per hour (gold standard for activity) and PROMIS® Physical Function and Fatigue short-form PROs in cancer patients receiving chemotherapy. These data suggest the utility of these PROs as tools for oncologists to improve their assessment of performance status, especially for male patients. Female patients had lower levels of steps/hour than males and lower correlations with PROs, suggesting the need for further work to improve performance status assessment in women.

## Data Availability

The datasets used and/or analyzed during the current study are available from the corresponding author on reasonable request.

## Abbreviations

CI: Confidence interval
ECOG: Eastern Cooperative Oncology Group
PRO: Patient reported outcome
PS: Performance status
